# Employees’ peak experience at work: Understanding the triggers and impacts

**DOI:** 10.3389/fpsyg.2022.993448

**Published:** 2022-10-20

**Authors:** Xiehong Fu, Jingru Ma

**Affiliations:** Glorious Sun School of Business and Management, Donghua University, Shanghai, China

**Keywords:** peak experience, proactive behavior, employees’ word-of-mouth referrals, high-activated positive mood, employee experience, grounded theory, affective events theory

## Abstract

The importance of providing a positive employee experience (EX) has gotten a lot of attention in recent years. However, peak experience (PE), as a highly positive experience, has been studied and applied in the field of human resource management only to a very limited extent. We still know little about how employees’ peak experience (EPE) happens and what the impact will be. Therefore, based on the affective events theory and the two-factor theory, our research conducted an in-depth exploration of EPE through three studies. In Study 1, we constructed a theoretical model centered on EPE based on and interview data. In Study 2, we developed and validated a scale for measuring triggers of EPE, which is a four-dimensional scale (elevation, insight, pride, and connection) with 16 items. In Study 3, we adopted structural equation modeling (SEM) to examine the relationship between EPE and its triggers as well as its impacts using data from 424 valid questionnaires. Our research shows that elevation, insight, pride, and connection can trigger EPE; employees are more likely to have proactive behavior (PB) and word-of-mouth referrals after they have PE; and the more job-relevant the triggers are, the stronger the association between PE and PB is. Our research provides a reliable and effective measurement tool for scholars to study EPE, broadens the findings of PE and EX, and points out feasible measures for organizations to create EPE.

## Introduction

We often hear the slogan “Customer is God” in the business environment. However, since good customer experience (CX) is provided by employees, managers began to realize that employees are the most valuable asset of the organization, and their focus has gradually extended from CX to employee experience (EX). With the concept of “Employee is also God” gaining popularity, an increasing number of managers have joined in the wave of improving EX. Positive EX can make employees feel happier (Rasca, 2018) and more satisfied (Du Preez et al., 2017), which in turn contributes to increased employee engagement (Lemon, 2019) and loyalty (Han and Lee, 2020), and ultimately improves the performance of employees (Lechermeier et al., 2020). As stated by Yohn (2019), if an organization can manage EX with the same standards as CX, it will lead to a unique and sustainable competitive advantage.

Although a large number of studies have shown that positive EX can bring many benefits to organizations (Batat, 2022), little is known about how peak experience (PE), an extraordinary experience, influences employees’ behavior. PE is defined as the happiest moment, ecstatic moment, and moment of rapture (Maslow, 1961), as well as a kind of intense ecstasy or the highest happiness (Privette, 2001). Research on consumers has proven that despite being a somewhat rare experience, the prominence of PE remains largely unquestioned (Ball and Barnes, 2017), because these defining moments can determine a customer’s decision. In addition, peak touristic experiences, which are defined as an experience with the maximum intensity or a most memorable experience, have been explored in many ways (Quan and Wang, 2004; Li, 2021). Maslow (1971) has also indicated that most people could have PE. However, after the concept of PE was put forward, little attention has been paid to exploring it in the field of human resource management (HRM) compared with many studies in the fields of marketing and tourism. Carù and Cova (2003) also indicated that it is necessary to search for intense pleasures and high arousal rather than the tepid mediocrity of everyday life. This raises an interesting question: what is the meaning of PE for employees? Thus, our research will explore what employees’ peak experience (EPE) means for employees and organizations.

Research investigating EPE has either investigated the triggers of PE in a certain group of employees (Lanier et al., 1996; Kim and Wang, 2020) or focused on one specific way of, how to motivate civil servants by some measures based on PE (Sun, 2013). These studies have not examined the effectiveness of motivational measures, and we know little about what kinds of factors can create PE for a wider group of employees. Maslow (1971) also indicated that many events could trigger PE. Thus, if the ways to enable EPE are diverse, the first goal of our research is to find ways that enable different organizations to create this highly positive experience for their employees.

Despite the recognition that PE can be triggered by various factors (Laski, 1962; Maslow, 1971; Hoffman et al., 2021), research on PE largely involves the identification of categories of triggers with little emphasis on how to measure these triggers. To date, in the field of PE, it has yet to be explored how to measure the triggers of PE in their own right. Thus, the second goal of our research is to develop an effective scale to measure the triggers of EPE and use it to verify the theoretical model.

Moreover, previous studies have demonstrated that PE will impact people’s cognition and behavior (Maslow, 1961; Kim and Wang, 2020). The two-factor theory shows that motivational factors can promote employee behavior while hygiene factors can only ensure employees are not dissatisfied. Thus, although there may be many factors that can make employees have PE, if we have not identified the type of factor, we cannot know if all the positive emotions that employees have can encourage them to be more active. Therefore, the third goal of our research is to explore the difference in the impact of PE on employees, which is obtained in different ways.

In general, although scholars have long been interested in PE, less work has focused specifically on EPE. Our research contains three aspects of the theoretical contributions: We will indicate the triggers of EPE and solve the problem of how to measure these triggers. In addition, we take a step toward filling this void by developing a measurement scale, which is effective to be used in the future to further research EPE and promote the empirical research progress of EPE. We will also explore the impact of EPE and clarify what factors can motivate employees to be more active at work. Moreover, our research will shed new light on the finding of PE in the field of HRM and help organizations find effective ways to create PE for their employees in managerial implications.

## Literature review

### Employee experience

The concept of EX comes from CX and user experience (UX), and involves the application of marketing knowledge and design thinking methodology by human resource managers (Sivathanu, 2019). We agree with Plaskoff (2017) that EX is employees’ perception of all contact points (e.g., activities, behaviors, and procedures) they encounter in the organization. Specifically, EX is defined as the meaning, connection, appreciation, and influence found by employees when they interact with management, colleagues, customers, technology, physical environment, organizational values, and work (Rasca, 2018).

The focus of our research is especially on one kind of positive EX. Existing researchers have discussed the measures of creating positive EX from many aspects due to the benefits that positive EX can bring. Yildiz et al. (2019) pointed out that organizations creating a positive EX should consider at least four dimensions: communication, leadership, positive organizational culture, and human capital development opportunities. Flexible work styles (Chen and Fulmer, 2017) and telecommuting (Anderson et al., 2014) can enhance EX by bringing more job-related positive affective well-being to employees. Applying a diverse set of HRM-focused AI applications operant could enhance the overall EX (Malik et al., 2022). A positive working atmosphere is also crucial to creating a positive EX, employees will feel happy when they have leisure experiences such as workplace fun (Tang et al., 2017). Building a good work environment is an essential way to improve EX (Bai et al., 2019), because workplace design aesthetics can affect employees’ subjective well-being (Kirillova et al., 2018). Leadership and colleague relationships are also important for shaping positive EX, responsive leadership and employee connectedness have been proven can improve EX in this digital age (Dery et al., 2017). Organizations have implemented a variety of ways to improve EX. For example, Cisco adopted a collaborative approach to enable employees to directly participate in the design of the “perfect” EX (Meister, 2016); Google allowed employees to purchase products at a free or reduced price; and Pandora catered to diverse work needs by signing a personalized agreement with each employee.

According to the affective events theory (AET) (Weiss and Cropanzano, 1996), specific events in the workplace could trigger emotional responses that then influence employees’ attitudes and behaviors. Much research has been done to improve EX by focusing on traditional HRM measures. However, since positive EX is closely related to employees’ positive emotions, it is also possible to create a positive EX by focusing on positive work events that can trigger positive emotions. Scholars have made some research achievements in this area. For example, the affective events-emotions matrix proposed by Basch and Fisher (2000) using qualitative analysis well relates work events and employees’ emotions. They found that events related to employees’ enthusiasm include goal achievement, involvement in planning, receiving recognition, coping with a challenge, and acts of colleagues. In addition, task-related success and positive feedback have also been shown to be associated with enthusiasm (Ohly and Schmitt, 2013). Since PE is often accompanied by positive emotions, our research aims to identify what kinds of work events can trigger PE using the framework of existing research.

### Peak experience

The concept of PE was originally introduced by Maslow, who defined it as “a sensory and perceptual experience, usually brief and profound, accompanied by enhanced perception, appreciation, and comprehension” (Maslow, 1964) as well as “the moment of the highest happiness and fulfillment” (Maslow, 1971). Research on positive human experiences also points out that PE is a highly positive feeling (Privette, 2001), defining it as an intense and highly valued moment. Therefore, we consider EPE, a highly positive feeling, as the moment of the highest happiness and fulfillment in employees’ work experiences.

According to the dimensional model of emotion, core affect is considered to be composed of two dimensions: pleasure (pleasure-displeasure) and arousal (activation-deactivation) (Russell, 2003). Positive mood can be further divided into high-activated positive mood (e.g., enthusiastic, inspired, excited) and low-activated positive mood (e.g., relaxed, laid-back, at ease) (Madrid et al., 2014). When people feel ecstasy or the highest happiness, they are full of energy and in a state of arousal. Thus, PE is a high-activated positive mood in essence.

Previous research has made significant progress in identifying the factors that trigger PE. For example, “art, nature, sexual love, religion, exercise and movement, creative work, “beauty,” childbirth, scientific knowledge, recollection and introspection, and poetic knowledge” were all common ecstasy triggers (Laski, 1962). Maslow (1971) also pointed out that music and sex are the most typical triggers of PE. These studies did not limit the occupation of participants, so subsequent research that explored PE focused on certain samples, such as sportsmen (Hollander and Acevedo, 2000), artists (Yeagle et al., 1989), backpackers (Ryan et al., 2003), and college students (Hoffman et al., 2021). Given the increased attention on EX, the goal of our research is to look deeply into the triggers that influence EPE in the workplace.

Existing studies have made important contributions to EPE. Lanier et al. (1996) found that realtors commonly reported that death, crisis, and spirituality were the triggers of their PE and that there was no difference in realtors’ recognizing and describing PE with others (e.g., students and artists). This research also indicated that PE can be extended to the workplace, which provides support for us to explore EPE. Kim and Wang (2020) proposed that office workers can have PE when they “fall into work with fun and interest” or “discover the self-existence at work”, and PE was considered as “a rudder of career development” and can “expand capabilities centered on oneself.” This research explored PE based on the employees’ career development experiences, which raises questions about what interesting findings can be found by exploring PE based on the employees’ normal work experiences. Sun (2013) indicated that means such as spirit inculcation, common vision, serious thinking, shaping culture precipitation, generating insight, and consistent self-inspection can make civil servants reach the PE moment. However, this research merely put forward some means to motivate civil servants based on PE, it did not use data to verify the effectiveness of these means. Moreover, previous studies have proven that PE is linked to eudaimonic as well as hedonic well-being (Whitehead and Bates, 2016) and has an impact on people’s attitudes on life (Hoffman et al., 2021). Thus, with the great benefit that positive EX can bring, it is surely warranted to further explore the motivational triggers and impacts of EPE, which is one kind of highly positive EX.

In addition, the book “The Power of Moments” published in 2017 by Heath and Heath, stated that all defining moments have one or more of the following four elements: elevation (rising above the ordinary), insight (rewriting one’s understanding), pride (capturing one’s best), and connection (feeling bonded with others). These elements can be used to design PE for customers, employees, or even patients. Moreover, Laws (2020) indicated that critical moments not only have plasticity, which means they can be created in different scenes, but also robustness, which means they can be recognizable because they can be identified by their core features. Therefore, based on the four-element conceptual model proposed by Heath and Heath (2017), organizations can take measures to make employees feel emotionally high if they can make use of the above elements.

Therefore, we will further explore the triggers and impacts of EPE in work environment. In addition, despite the existing findings of EPE being highly commendable, effectively verifying the theoretical model that is centered on EPE remains an unsettled issue. Thus, we will develop and validate a scale that can measure the triggers of EPE effectively and then use this scale to validate the theoretical model. Our research will not only enrich the findings of PE in the field of HRM but also help organizations discover the implementable measures of creating EPE.

## Overview of studies

Three studies were conducted to achieve the research goals. Study 1 constructs a model focusing on the triggers and impacts of EPE by using grounded theory. Study 2 develops and tests a scale for measuring EPE triggers. Study 3 uses the newly developed scale to test the theoretical model proposed in Study 1.

## Study 1

### Methodology

As one of the mainstream qualitative research methods, grounded theory is used to explore those phenomena or processes that are not fully understood (Corbin and Strauss, 2015). This method was proposed by Strauss and Glaser in 1967, and its core goal is to construct a new theory or conceptual proposition by collecting and analyzing qualitative data. This study follows the proceduralized grounded theory paradigm for data analysis, in which the basic steps of analysis include open coding, axial coding, and selective coding. In this study, we begin by collecting stories about PE and analyzing them to obtain initial concepts, then consider the relationship between these initial concepts, and finally construct a theoretical framework based on theoretical saturation.

### Sample

This study followed the method of theoretical sampling, which is a concept-based or topic-based data collection strategy that tries to fully develop concepts, explain their meanings, and establish a substantive theory (Liu and Li, 2017). During the process of sample selection, we took into account several criteria. First, we chose interviewees who had 1 year or more of work experience, as this enabled us to gather more useful information regarding the EPE. Second, we chose interviewees from a variety of industries and occupations as possible to increase the generalizability of the findings. Third, the sample size for this study is determined by theoretical saturation, which is the point where significant categories are not gained by adding more samples. Generally, we interviewed a total of 21 participants between December 2021 and January 2022, including 9 males and 12 females, whose ages were between 25 and 40. Their occupations included human resources, marketing director, secretary, sales, developer, and finance, and they had worked for 1–20 years, with positions ranging from general staff to senior management. Table 1 provides an overview of the 21 interviewees in this study. In addition, this study also investigated 26 MBA students using the same questions through an open-ended questionnaire to verify the results from semi-structured interviews, including 10 males and 16 females, whose working years ranged from 1 to 25 years.

**TABLE 1:** Sample basic information of interviewees.

No.	Gender	Education	Tenure	Occupations	No.	Gender	Education	Tenure	Occupations
1	Female	MBA	12	Chairman secretary	12	Male	MBA	14	Human resource
2	Male	MBA	2	Entrepreneurs	13	Male	MBA	6	Finance staff
3	Male	MBA	1.5	Office assistant	14	Female	MBA	12	Headhunter
4	Female	Master	1	Human resource	15	Female	Master	1	Human resource
5	Female	MBA	16	Human resource	16	Male	MBA	19	Equipment management
6	Female	Master	1.5	Human resource	17	Male	MBA	7	Technical leader
7	Male	MBA	12	Software developer	18	Female	MBA	10	Human resource
8	Male	MBA	3	Sales	19	Male	MBA	12	General director
9	Female	MBA	3	Student administrator	20	Female	MBA	20	Human resource
10	Female	MBA	6	Account manager	21	Female	MBA	7	Marketing director
11	Female	MBA	8	CDC section member					

### Data collection

To increase the validity and reliability of our interviews, we developed an interview outline before the semi-structured interviews, which focused on the core questions of “triggers of EPE” and “impacts of EPE.” The interview outline is the overall framework of the interview content, the researcher could maintain flexibility by adjusting questions based on the interviewee’s statements during the actual interview.

Each interview began with an overview of the interviewees’ education, tenure, and job. With the consent of the interviewees, we recorded all the contents during the interview. After interviewees introduced the basic contents of their work, they were asked to choose a specific period to draw a graph of their true emotional changes at work. Then interviewees are encouraged to describe the story at the emotional curve’s peaks (including when, where, why, and how, as well as elements such as sensory experiences and emotions at that time), followed by more probing questions about this work event. In addition to the details of when the event occurred, interviewees were also asked to talk about how it affected their behavior. After the formal interview, we utilized Sogou dictation to convert the audio recordings into written materials, and we read interview transcripts multiple times, asking participants for clarification (where necessary). Finally, we used NVivo to independently analyze all of the interview materials, then discussed the themes and items and achieved consensus.

In this study, 21 personal in-depth interviews were conducted successively with the theme of “triggers and impacts of EPE” and each interview lasted about 30 min. By the time we reached 18 interviews, we discovered that the current theoretical framework had captured the majority of the data. Nevertheless, we continued to interview, keeping an open mind to any new concepts. We recorded the interview data of roughly 170,000 Chinese characters after interviewing 21 interviewees and discovered that the last three interview materials did not reveal any novel essential categories or theoretical relationships. Furthermore, the theoretical framework constructed based on the interview data is also confirmed and supported by the findings of Heath and Heath (2017). Questions in the interview outline were also investigated using an open-ended questionnaire in this study, which once again verifies the theoretical framework. Therefore, we believe that the theory has reached saturation.

### Analysis

#### Open coding

Open coding is the first step in the analysis of interview data, where the main purpose is to refine original data and extract categories. This step started with a careful reading of the interview data. We have an open mind when coding the interview data word by word and refining significant concepts for each fragment (Pieterse, 2011). Some general questions were considered to help in this process. For instance, what is happening in the data fragment? Or, what does the data fragment express? To minimize errors and subjective biases, the initial concept was named as closely as possible in the interviewees’ own words. In this study, three graduate students in the field of PE coded all the interview data individually and kept their analytical diaries. Upon completion of the independent analyses, students met and discussed under the guidance of the instructor. When disagreements emerged, we were fully communicating and achieving an agreement to ensure coding accuracy. According to the Holsti reliability coefficient formula, the final reliability of this study is 0.88, indicating that the coding results have high reliability. Through the analysis of the original data in this stage, 61 initial concepts (a1–61) are extracted and further divided into 22 categories (A1–22). Table 2 shows some of the initial concepts and categories.

**TABLE 2:** Examples of open coding.

Category	Initial concept	Interview data direct quotations from participants
A4 sense of unexpected surprise	a10 achieving beyond expectations	Some of the contents in the discussion they output are beyond my imagination.
	a29 unexpected successes	In my subconscious, I know that the rate of success is very low, so I am extremely happy when I get this unexpected result.
A9 gain growth through reflection and insight	a14 opportunity for growth after self-reflection	I changed my way of doing things. When I encounter problems again, I only ask others when they cannot meet customer needs.
	a35 moments of comprehending opportunity	I know that this is an opportunity the company provides for employees’ development and growth, which makes me emotionally high.
A12 pride after being recognized	a13 professionalism recognized by others	If a candidate praises you and thinks you are professional, it means you are recognized.
	a18 highly appreciated by customers	Anyway, he thought I was trustworthy, and being recognized by customers is definitely a high evaluation for me.
	a25 received late recognition	When I resigned, my colleagues expressed their sadness to me. At that time, I felt that my serious work attitude was recognized.
	a26 received late recognition	I found that some of the people whom I selected for the organization also got recognition, which makes me very happy.
	a39 recognized by leaders and subordinates	In fact, what makes us happy is that we can help the team solve problems and get their recognition.
	a43 strength was proven and confirmed	That is to say, I did not live up to my leadership, and it is also a kind of affirmation of my strength.
A15 warmed by caring and mutual help	a12 feeling humanistic care from the company	The company is very considerate to provide some things, especially for us, and these things reflect high humanistic care.
	a28 perceived enthusiastic help from colleagues	It should be that when I just started my work, I found that my colleagues were very friendly to me and they helped me quickly adapt to this new work.
	a33 received care from an approachable executive	He asked me which department I belonged to, how I was feeling, whether I was tired from work, and other thoughtful questions.
	a40 humanistic care obtained at the beginning of employment	The person in charge of recruitment came to care about my recruitment progress, gave me a bunch of flowers, and expressed her welcome.
A18 be proactive about work	a55 more proactive work behavior	Mutual cooperation and help could promote a positive change in our work attitude.
	a57 willing to spend more energy on work	The atmosphere of moving forward toward the same goal will certainly affect my work attitude. It means that I will be willing to spend more personal time on my work.
	a60 more proactive in approaching work	I will take control of this project more seriously and proactively. If there is a problem, I will take the initiative to solve it.
A21 genuinely share work experiences	a53 willing to actively share positive information about organizations	I have a lot of recognition for this organization, and I will take the initiative to share positive information about the organization.
	a58 share positive experiences of organizations on social network	I showed my friends the flowers sent by the person in charge of recruitment and the scene of my coworkers welcoming me on WeChat.

#### Axial coding

Axial coding is a further analysis of interview data based on open coding, aiming to extract main categories from existing categories. This stage focuses on organizing related categories around an “axis”, establishing linkages between categories (Juliet and Strauss, 2017), and summarizing them into main categories, which are more concentrated. During this process, three graduate students determine the relationship between categories and main categories independently, as well as classify and reorganize categories according to the levels, dimensions, and characteristics of main categories. Students also met and discussed when they finish their work. In addition, to avoid excessive complexity, some categories were merged or deleted as needed after a full discussion. The results of axis coding are summarized in Table 3. These 22 categories respectively reflect the dimensions and characteristics of the six main categories [elevation, insight, pride, connection, proactive behavior (PB), and employees’ word-of-mouth (WOM) referrals]. They can be split into triggers and impacts based on the relationship between main categories and EPE.

**TABLE 3:** Axial coding.

Main category	Category
AA1 elevation (moments of transcending ordinary events)	A1 excitement of finally getting what we want
	A2 freshness of work change
	A3 pleasure of reward for diligence
	A4 sense of unexpected surprise
	A5 satisfaction of receiving high-value benefits
	A6 feelings of relief
	A7 sense of luck in receiving special treatment
AA2 insight (moments of rewiring one’s understanding)	A8 make a breakthrough and demonstrate one’s potential
	A9 gain growth through reflection and insight
AA3 pride (moments of capturing one’s best)	A10 sense of mission given by work
	A11 sense of achievement that reflects personal value
	A12 pride after being recognized
	A13 the honorable feeling of not living up to trust
	A14 sense of superiority
AA4 connection (moments of being bonded with others)	A15 warmed by caring and mutual help
	A16 work together toward the goal
AA5 proactive behavior	A17 bravely face challenges
	A18 be proactive about work
	A19 self-directed learning for advancement
	A20 take responsibility to help the organization
AA6 employees’ word-of-mouth referrals	A21 genuinely share work experiences
	A22 sincerely recommend the company

#### Selective coding

**FIGURE 1:**
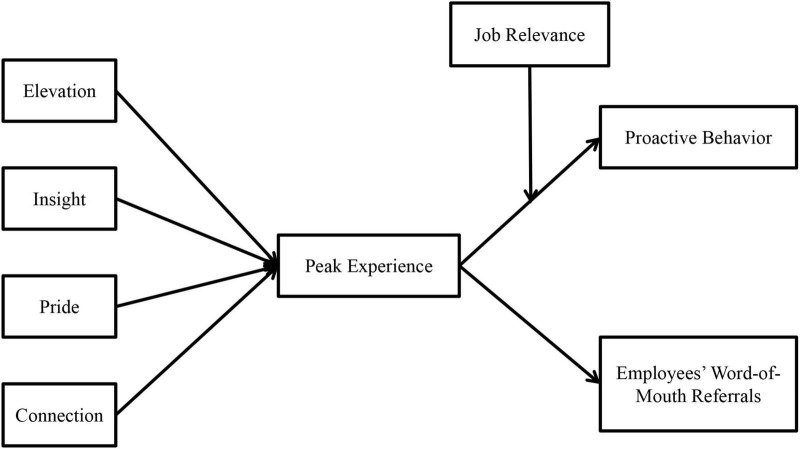
Theoretical model.

The goal of selective coding is to systematically organize data and construct a theoretical model. According to the purpose of our research, the core category is “triggers and impacts of EPE.” Thus, we use the core category as the central for incorporating relevant main categories into the theoretical model through the storyline. Specifically, we organize the dispersed main categories, identify their relationships, and integrate them into the core category. Finally, a theoretical model centered on EPE and connecting the main categories was constructed, which is shown in Figure 1.

#### Model interpretation and hypotheses

This study constructs a theoretical model around EPE based on AET through a series of coding. This model mainly explains two issues. On the one hand, it identifies four triggers that lead to EPE: elevation, insight, pride, and connection. On the other hand, it clarifies that employees’ WOM referrals and PB are more likely to occur after employees have PE. These four triggers correspond to four types of work events, which can be individually or jointly used to create PE for employees. As a highly positive emotion, PE can affect employees’ attitudes and promote two types of affect-driven behaviors. The following section of this study will explain each main category.

#### Elevation

Elevation refers to the moments that transcend the normal course of events (Heath and Heath, 2017), it can provoke not just transient happiness, but memorable delight. Through the analysis of interview data, it can be found that elevation can occur at all stages of the career journey. When employees receive a job offer from the company they are looking forward to, they will feel very excited because their long-standing wishes are finally being met. The freshness brought by a change of work is also rarely experienced in daily work. Whether it is the change from the campus environment to the work environment or starting a new job, both of these represent a milestone in the employee’s career. In the process of completing a task, employees need to make continuous efforts. When they finally achieve the goal, they will be very joyful. Workplace entertainment is especially vital for improving employees’ emotions who are becoming increasingly stressed. Employees can also be emotionally high by participating in activities with an exciting atmosphere, such as annual meetings, sports games, and parties. Furthermore, if employees are given particular privileges, such as greater autonomy, they will feel unique and lucky, thus resulting in positive emotions.

Some studies have paid attention to the relationship between these work events and positive emotions. According to the expectancy disconfirmation theory, a highly satisfying emotion will occur when an employee’s actual perception of the experience exceeds the expected level (Magnini et al., 2011). If organizations can let their employees have an out-of-ordinary experience, such as gaining a “surprise bonus”, it will raise the employees’ emotional level (Engellandt and Riphahn, 2011). Having high-value benefits is also important for influencing emotions, the higher the level of salary employees receives, the more likely they will have positive emotions (Koo et al., 2015). Work autonomy represents the level of trust employees receive and a high level of work autonomy can also boost positive emotions (Wegge et al., 2006). Leisure experience has been recommended as a means by which to trigger positive moods and emotions (Tavel et al., 2022). Previous research has indicated that sedentary adults can experience positive emotions during leisure because leisure time physical activity environment seems highly motivational for them (Harikkala-Laihinen et al., 2022). From the definition and existing research, we can find that there is a close association between elevation and employees’ positive emotions. Considering the essence of PE is a highly positive feeling, we propose the following hypothesis:

H1a: Elevation has positive effects on PE.

#### Insight

Insight refers to the moments that rewire one’s understanding of themselves or the world, which might influence their lives for decades (Heath and Heath, 2017). According to the interview data, these work events include making a breakthrough and demonstrating one’s potential, as well as gaining growth through reflection and insight. The former refers to an employee completing work that was previously thought to be unattainable. Such events make employees realize their potential in the process of constantly breaking through themselves, thus affecting their attitude toward difficulties. The latter mainly focuses on growth, which can come from reflection at work. In either case, this growth will have a significant impact, such as changing how employees think or act. Employees can naturally experience positive emotions when they recognize the significance of such events.

This viewpoint can also be supported by existing findings. The incident of inspiration is an experience of suddenly discovering new realizations and insights, and two states of inspiration experience, inspired-by and inspired-to, have an influence on emotional responses of delight and transcendence (Khoi et al., 2021). PE is a kind of high-activated positive mood, so we think employees will get PE in the process of having a deep understanding. Furthermore, humans are inherently eager to develop and grow to their full potential (Deci and Ryan, 2000), and more than half of PE were related to learning and growing experiences, according to Mouton and Montijo (2017). Thus, we hold the point that employees’ emotions will be positive if employees can obtain the insight to show their potential or promote their growth. Based on the above analysis, we put forward the following hypothesis:

H1b: Insight has positive effects on PE.

#### Pride

Pride refers to the moments that capture one at their best (Heath and Heath, 2017), it is a celebration of an employee’s achievements as well as an action that demonstrates their value. According to the interview data, when employees realize the significance of their work, such as bringing happiness, solving important problems, or making meaningful contributions, they will have a sense of pride. Employees can demonstrate their value to others and keep their emotions high by being the first to achieve performance goals. Among those events that can trigger EPE, the highest proportion is recognized by others. This recognition can come from customers (e.g., customers praise an employee’s professionalism), leaders (e.g., leaders praise an employee’s ability), and can also be reflected in various work events (e.g., an employee wins the trust of leaders).

The majority of positive work events stated by respondents in existing research are related to pride. Praise, appreciation, and positive feedback from others account for a large proportion of positive events, and so do events connected to task completion, such as effectively solving problems (Ohly and Schmitt, 2013). Events where employees can prove their abilities and demonstrate their potential are also a kind of pride (Grandey et al., 2002). According to Dormann and Zapf (2004), employees using their skills to help customers efficiently solve problems can improve their sense of accomplishment, which is generally accompanied by positive emotions. In other words, being able to help customers solve problems is an event that can easily trigger positive emotions for employees (Kiffin-Petersen et al., 2012). Tavel et al. (2022) also indicated that a person was pleased with themselves for handling a challenging situation and enjoying it. In addition, Eisenberger et al. (2005) found that achievement-oriented employees are often in a high mood when facing highly skilled and challenging tasks because successfully completing these tasks can demonstrate their values, which suggests that pride is associated with a high mood. Our research considers PE is a high-activated positive mood, thus we propose the following hypothesis:

H1c: Pride has positive effects on PE.

#### Connection

Connection refers to the moments when being bonded with others (Heath and Heath, 2017). These types of events can be separated into two categories based on the interview data. One category is warmed by caring and mutual help, which emphasizes caring and warmth. Employees’ emotions can be stimulated by the concerns of colleagues and leaders, such as meticulous care from colleagues while taking over a new job and timely help from leaders when encountering difficulties. Perceived humanistic care will also make employees realize the organization is so considerate. The other category is working together toward the same goal, which emphasizes unity. In the process of all team members working together to face challenges and solve problems, everyone unites and helps each other, this high cohesion can make emotions high.

We can also find support for the above view from the existing literature. Positive feelings can be generated by colleagues’ help, support, and other friendly behavior (Basch and Fisher, 2000). These pleasant interpersonal interactions make individuals’ relationships more intimate and affect employees’ positive emotional states (Dimotakis et al., 2010). Employees’ emotions can also be influenced by the team climate. Music festivals, for example, can produce a collective enjoyable experience, and emotional contagion makes these positive feelings permeate (Wood and Kinnunen, 2020). In the problem-solving process, the team is filled with a positive atmosphere of mutual help and unity, employees will naturally feel happy when they recall such events. Research on tourism experiences has proven that companionship plays a key role in shaping memorable tourism experiences (Vada et al., 2022). This episode is remembered mostly because it exceeded a certain level of emotional arousal (Mitas et al., 2020). Thus, we can infer that companionship is essential for EPE, which is a highly positive emotion. Based on the above analysis, we propose the following hypothesis:

H1d: Connection has positive effects on PE.

#### Proactive behavior

Proactive behavior (PB) is the active, future-oriented behavior of employees in the workplace (Grant and Ashford, 2008; Parker et al., 2010), as well as spontaneous behavior that goes beyond their job roles. Interview data shows that after having PE, employees’ work attitudes will be altered, and they will be more proactive at work. When employees finally solve a problem through hardships, their ability can be proven, which will encourage them to be more active when facing challenges in the future. The atmosphere of cooperation and mutual help in the team will lead employees’ emotions at a high level, making them more positive toward work. Employees will also be willing to take the initiative to continuously improve themselves through self-learning in order to meet their motivational requirements, obtain acknowledgment from others, and feel a sense of accomplishment. When employees have positive experience in the organization, they will firmly establish a sense of ownership, be more responsible for their work, and try their best to help the organization achieve its goals.

Emotions have been widely recognized as an important factor influencing employees’ behavior (Diener et al., 2020). The more positive an employee’s perceived emotion is, the more time they will spend on PB (Fay and Sonnentag, 2012), which means a high level of positive emotion is conducive to employees’ positive behaviors. Furthermore, positive emotions, according to the broaden-and-build model (Fredrickson, 2001), can be used as a resource to motivate employees to work hard to achieve goals (Parker et al., 2010). Researchers have indicated that a high-activated positive mood can provide significant motivational power for enabling employees to have a high level of energy, spirit, and enthusiasm (Boudrias et al., 2022), as well as stimulating individual effort (Gable and Harmon-Jones, 2010). Moreover, Mouton and Montijo (2017) indicated that passion and PE share definitional and theoretical overlaps, and previous research has proven that high-intensity emotions (e.g., passion) can lead to PB (Mindeguia et al., 2021). Thus, it can allow employees to show more excitement at work (Bindl et al., 2012) and make them more willing to engage in various forms of PB (Wang et al., 2019). Therefore, our research wants to test whether PE, which was considered as a high-activated positive mood essentially, could play the same role in driving employees to engage in PB. Thus, we put forward the following hypothesis:

H2a: PE has positive effects on employees’ PB.

#### Employees’ word-of-mouth referrals

Employees’ WOM referrals are a process in which employees and former employees exchange information and opinions about the organization both inside and outside the social network (Keeling et al., 2013). Interview data shows that when employees have positive EX, they are more willing to actively disseminate positive information about the organization with others and share the positive experience they had on their WeChat. Positive EX also makes employees willing to take advantage of various opportunities to recommend their organization to others, and the essential reason for this out-of-role behavior is that employees truly enjoyed a positive experience in the organization.

Affective events theory (AET) (Weiss and Cropanzano, 1996) points out that individual emotions will affect individual behavior, such as organizational citizenship behavior (OCB) (Spector et al., 2010). Existing research has proven that daily mood changes are associated with OCB (Clark et al., 2018). According to Xu et al. (2020), employees’ WOM referrals are kinds of OCB, which means that employee emotions will affect employee WOM referrals. Studies have also shown that positive emotions are more likely to promote the spread of WOM (Septianto and Chiew, 2018), and those positive high-intensity and arousal messages that can lead to positive emotions (e.g., delight, interest, surprise, and joy) are more likely to be shared (Heath et al., 2001). Li (2021) indicated that if tourists have PE, these remarkable experiences will further influence their recommended intentions. Mitas et al. (2020) also found that participants who are highly recommended because their emotions arouse a peak during tourism, this peak is higher than the emotion level before and after the tourism. This raises an interesting question that will employees’ WOM referrals be influenced if they have PE? Moreover, high-activated positive mood has been shown to have a driving effect on employee behavior (Seo et al., 2010). In addition, the PE that our research focuses on is a high-activated positive mood, so we have reason to presume a causal relationship between PE and employees’ WOM referrals. Thus, we put forward the following hypothesis:

H2b: PE has positive effects on employees’ WOM referrals.

#### Moderation effect of job relevance

The two-factor theory proposed by Herzberg divides the factors that affect employee behavior into two categories: motivational factors and hygiene factors. Hygienic factors such as interpersonal relationships and working conditions are usually unrelated to the work itself. On the contrary, motivational factors are largely related to the work itself, such as the challenge of the work, the sense of achievement brought by the work, and the importance of the work. In our research, we define job relevance (JR) as the relevance of triggers of EPE to the job. Specifically, if the triggers are largely related to the work itself, it means high JR. If the triggers are largely unrelated to the work itself, it means low JR. According to the two-factor theory, motivational factors can stimulate employees’ enthusiasm and make them generate excitement and energy (Liu et al., 2021), thus making employees proactive at work. While analyzing the interview data, we also found evidence that supports this viewpoint. When employees have successfully obtained the qualification to execute a project after going through difficulties, they will always maintain a serious and responsible attitude in the subsequent implementation process and take the initiative to solve the problems they encounter. Therefore, we think that when the factors that trigger EPE are mostly job-related, employees will more likely have PB. In other words, the higher the JR, the closer the relationship between PE and PB. Based on the above analysis, we put forward the following hypothesis:

H3: JR moderates the relationship between PE and employees’ PB.

#### Mediation effect of peak experience

Elevation refers to an extraordinary moment beyond the ordinary, in which behavior will be affected. Scholars in the field of marketing have found that positive WOM referrals are more likely to be driven when customers’ actual experience exceeds expectations (Ludwig et al., 2017), and the mechanism of influence is the positive emotion generated by such a surprise. We can extend this scenario to employees. Employees’ positive emotions are also conducive to the occurrence of WOM recommendations when the actual experience they perceive as exceeding expectations. In addition, when customers can obtain positive emotions in their experience, it can promote the occurrence of their repurchase behavior. Similar to how customers are motivated, employees might become more proactive in their work when they have great emotional experiences at work. Therefore, we believe that PE plays a mediating role between elevation and employees’ PB or employees’ WOM referrals.

Employees might learn more about themselves and uncover their potential when they experience the “moment of insight”. A deeper understanding of one’s potential enables employees to appreciate that they have the ability to do so, thus giving them the courage to face difficulties in the future. Goal-oriented employees consider challenging work tasks as a way to improve themselves because they know that completing highly challenging jobs would satisfy their need to learn and develop. Goal-oriented employees are more likely to have a high-activated positive mood when dealing with these really difficult activities (Preenen et al., 2014), and PE is a high-activated positive mood. Additionally, we think that goal-oriented workers will be eager to share experiences with others that help improve their skills. Therefore, we believe that PE plays a mediating role between insight and employees’ PB or employees’ WOM referrals.

A moment of pride is a time when employees can present themselves to others and earn praise and recognition from others. It has been pointed out in previous studies that recognition and praise from others can help employees work harder. In semi-structured interviews, it was also pointed out that recognition from customers can make employees more serious and proactive in their work, and that recognition from leaders can certainly make employees dare to actively solve problems. We believe that the reason why employees have such behavior is that such recognition and praise make their emotions positive. In other words, employees get PE in this process. This high level of positive emotion is conducive to employees’ positive work behavior. Employees in organizations where they can earn praise and rewards are also more likely to suggest and share their organizations with others since they feel more good feelings in such an environment. Therefore, we believe that PE plays a mediating role between pride and employees’ PB or employees’ WOM referrals.

A moment of connection is a time when employees can feel connected to other members of the organization. When a team is faced with a difficult but important task, the team members help each other and work together toward the same goal to successfully complete the task. Employees’ feelings have been strengthened as a result of this event, which will help them recognize how interdependent and inseparable they are from other team members. When they realize this, the emotion is positive, which allows them to be more proactive in their work later on. In addition, Wood and Kinnunen (2020) pointed out that collective behavior or co-creation behavior will make collective emotional memory more profound, and the team behavior of solving problems together is a collective behavior. In this process, the team is permeated with a positive emotional atmosphere of mutual help and solidarity. Employees will naturally feel happy when recalling such events and will also be willing to recommend this type of organization to others. Therefore, we believe that PE plays a mediating role between connection and employees’ PB or employees’ WOM referrals. Based on the above analysis, we put forward the following hypotheses:

H4: PE mediates the relationship between four triggers and two behaviors.

H4a: PE mediates the relationship between elevation and employees’ PB;

H4b: PE mediates the relationship between elevation and employees’ WOM referrals;

H4c: PE mediates the relationship between insight and employees’ PB;

H4d: PE mediates the relationship between insight and employees’ WOM referrals;

H4e: PE mediates the relationship between pride and employees’ PB;

H4f: PE mediates the relationship between pride and employees’ WOM referrals;

H4g: PE mediates the relationship between connection and employees’ PB;

H4h: PE mediates the relationship between connection and employees’ WOM referrals.

## Study 2

### Methods

In this study, a scale for measuring the triggers of EPE will be developed and validated using data from interviews. Specifically, the process of developing the scale includes the following steps. First, define the concept of each trigger. Second, develop the initial item pool and invite experts to optimize the items. Finally, collect the questionnaire data to conduct exploratory factor analysis (EFA) and confirmatory factor analysis (CFA) and test the reliability and validity of the scale.

The successful development of this scale has two meanings. On the one hand, this scale can be used to measure the extent to which these events can make employees’ emotions rise to peak instantly. After having a clear understanding of these events, organizations can design positive experiences for employees. Previous research, on the other hand, has identified some factors that can make employees feel positive, but there is no instrument to assess triggers. Therefore, a scale should be developed to further investigate the triggers of EPE.

### Definition

Our research finds that the factors that trigger EPE include “elevation”, “insight”, “pride”, and “connection” after analyzing interview data. Specifically, elevation refers to the moments that let employees experience out-of-the-ordinary work events. Insight refers to the moments that rewrite their understanding of themselves or the world. Pride refers to the moments that capture their best. Connection refers to the moments that make employees feel bonded with others.

### Item generation

Study 2 aimed to generate items based on semi-structured interviews. Previous literature pointed out that events such as goal attainment, problem-solving, praise, social interactions, and receiving recognition are related to employees’ positive emotions (Basch and Fisher, 2000; Ohly and Schmitt, 2013). Our research also collected some work events about the triggers of EPE through interviews, and the items came from interview data.

In general, we generated an initial item pool of 21 items in four subscales: “elevation”, “insight”, “pride”, and “connection.” In addition, in order to ensure the consistency and rigor of the scale, experts in the field of HRM were also invited to review and modify items. This also ensures the authority and rationality of the items, as well as content validity and surface validity. All of these items use the seven-point Likert scale, and the degree of agreement ranges from “very disagree” to “very agree”, with scores of 1–7.

### Sample and procedure

Participants on social networking and Credamo were invited to participate in the study in April 2022. They were invited to participate voluntarily in the online survey and were told that this survey aimed to investigate their work experience over the past month. First, the definition of PE was shared with the participants. Then, the question “Do you have work experience in the past month?” was used to identify participants because they are required to recall the work events and feelings from their past work experience. Only those who had work experience in the past month were suitable to finish the survey. As a result, a total of 325 questionnaires were returned to the researchers, of which 46 were eliminated because the answer time was less than 100 s or the trap questions were not answered correctly, and 280 valid data were finally obtained with effective recovery rates of 86.15%. This part of the data was named Sample 1, and the key characteristics of this sample are as follows. The total number of female participants is 62.86%, compared to a total of 37.14% males. Qualifications of participants ranged from undergraduate degree holders (73.57%), master’s degree and above holders (18.93%), to only 7.5% with others. In terms of tenure, 33.57% of participants worked fewer than 1 year, 17.50% of participants worked 2–3 years, 19.29% of participants worked 4–6 years, 15.71% of participants worked 7–9 years, and 13.93% of participants worked more than 10 years. Table 4 shows the demographic statistics of Sample 1.

**TABLE 4:** Demographic characteristics of Sample 1 and Sample 2.

Gender		Age		Education		Tenure	
**Sample 1**							
Female	62.86%	≤ 25	38.57%	High school and below	0.71%	≤ 1 year	33.57%
Male	37.14%	26–30	31.07%	Associate degree	6.79%	2–3 years	17.50%
		31–35	18.93%	Bachelor’s degree	73.57%	4–6 years	19.29%
		≥ 36	11.43%	Master’s degree and above	18.93%	7–9 years	15.71%
						≥ 10 years	13.93%
**Sample 2**							
Female	59.20%	≤ 25	31.13%	High school and below	0.47%	≤ 1 year	26.18%
Male	40.80%	26–30	33.25%	Associate degree	4.95%	2–3 years	17.92%
		31–35	22.41%	Bachelor’s degree	75.71%	4–6 years	20.75%
		≥ 36	13.21%	Master’s degree and above	18.87%	7–9 years	19.10%
						≥ 10 years	16.04%

To test the factor structure and verify the fit between data and model, 507 questionnaires were collected in the same way in May 2022, of which 83 were eliminated because the answer time was less than 100 s or the trap questions were not answered correctly, and 424 valid data were finally obtained with effective recovery rates of 83.63%. This part of the data was named Sample 2 and the key characteristics of this sample are as follows. The total number of female participants is 59.20%, compared to a total of 40.80% males. Qualifications of participants ranged from undergraduate degree holders (75.71%), master’s degree and above holders (18.87%), to only 5.42% with others. In terms of tenure, 26.18% of participants worked fewer than 1 year, 17.92% of participants worked 2–3 years, 20.75% of participants worked 4–6 years, 19.10% of participants worked 7–9 years, and 16.04% of participants worked more than 10 years. Table 4 also shows the demographic statistics of Sample 2.

### Analysis

#### Exploratory factor analysis

We used SPSS 26.0 to analyze the data from Sample 1. The KMO was 0.893 > 0.70 and the chi-square approximation of Bartlett’s test of sphericity was 1493.233 (df = 120, *p* < 0.001), which indicates that there are common factors among the items and the data is appropriate for using EFA. The varimax rotation is used for factor rotation to extract factors with eigenvalues greater than 1. Combined with the gravel plot, a total of four factors are extracted, named elevation, insight, pride, and connection.

The factor load should ideally be greater than 0.4 (Hinkin, 2005). In order to simplify the scale, we deleted four items with a factor load lower than 0.5. If a component factor contains items from several factors, we choose to retain the items that belong to the most factors, thus deleting one item. The final version of the scale contains 16 items (including 5 items for elevation, 4 items for insight, 4 items for pride, and 3 items for connection). The cumulative variance contribution rate is 59.61%, which meets the requirements of scale development. In addition, each dimension had 3–5 items, which means the scale was reasonable in terms of the number. The specific content of the items is shown in Table 5.

**TABLE 5:** Results of exploratory factor analysis (EFA).

Item	Factor			
	1	2	3	4
**Elevation**				
I have the authority for self-determination at work.	0.699			
I can actually enjoy the high-value benefits provided by the organization.	0.680			
I have experience of making a large amount of income at one time during work.	0.639			
I have work experience that I didn’t hold out hope for at first but got unexpected results finally.	0.598			
I feel interested when I recall the experience of participating in activities (e.g., annual meetings).	0.577			
**Insight**				
I have gained something that can change my way of doing things from this work.		0.815		
I discovered a new way of thinking through this work.		0.736		
I have benefited a lot from those self-improvement actions at work.		0.626		
I can appreciate that the organization provides me with the opportunity to grow and develop.		0.522		
**Pride**				
I think I have really contributed to the organization.			0.767	
I think I have lived up to the trust and support I received from the organization.			0.679	
I found that my serious attitude toward work was affirmed by my colleagues.			0.663	
My ability has been fully demonstrated through the completion of certain work.			0.593	
**Connection**				
When I first started this work, I could feel that everyone was helping me adapt.				0.797
When I first started this work, I could feel that everyone welcomed and cared for me.				0.729
I can feel everyone helping each other in this work.				0.714

#### Confirmatory factor analysis

In order to further confirm the structure of the scale, this study used the method of maximum likelihood estimation to conduct a series of CFA for different models by AMOS 26.0 using Sample 2, including a single-factor model, two-factor model, three-factor model, and four-factor model. We checked the model fit for each model using the fit indicators and the χ^2^/df, and found that all the alternate models exhibited significantly worse fit than model 1, the base model (Table 6). Thus, it shows that the design of the scale structure is reasonable. In addition, the standardized factor loadings between all observable variables and their corresponding latent variables are all greater than 0.5, indicating that the scale has good convergence.

**TABLE 6:** Indicators of model fit.

Model	χ^2^	df	χ^2^/df	Δχ^2^/df	RMSEA	CFI	GFI	IFI	TLI	RMR
Model 1	209.34	98	2.136		0.052	0.938	0.94	0.939	0.925	0.046
Model 2	298.669	101	2.957	89.329/3***	0.068	0.891	0.913	0.892	0.87	0.056
Model 3	304.107	101	3.011	94.767/3***	0.069	0.888	0.911	0.889	0.866	0.056
Model 4	348.924	103	3.388	139.584/5***	0.075	0.864	0.897	0.865	0.841	0.062
Model 5	383.166	104	3.684	173.826/6***	0.08	0.846	0.889	0.847	0.822	0.064

Model 1: four-factor model; Model 2: three-factor model (elevation, pride, insight + connection); Model 3: three-factor model (elevation, insight, pride + connection); Model 4: two-factor model (elevation + insight, pride + connection); Model 5: single-factor model.

***p < 0.001.

#### Reliability and validity analysis

To test the internal consistency of the scale, we calculated Cronbach’s alpha values for all four subscales, which ensures the reliability of the scale. It was found that elevation α = 0.717, insight α = 0.730, pride α = 0.688, connection α = 0.716, indicating that the scale had good content consistency as well as good reliability.

Validity analysis is used to test the accuracy of items. The convergent validity of the scale was tested by calculating the construct reliability (CR). The CR of four subscales was found to be higher than 0.60, indicating that the scale has better convergent validity (Hair et al., 2018). In addition, the scale also has good content validity, which means the items can truly reflect the concepts. There are three reasons for good content validity. First of all, the process of developing the scale follows a standardized procedure when carrying out semi-structured interviews and issuing questionnaires. Second, the coding process was conducted by several researchers. Finally, the initial items were obtained after several rounds of discussion among experts. Therefore, the development of this scale is scientific and rigorous. The specific content of the scale is shown in Table 7.

**TABLE 7:** Construct reliability (CR), Cronbach’s alpha, and correlations.

			Correlations						
Construct	CR	α	1	2	3	4	5	6	7
Elevation	0.728	0.717	1						
Insight	0.729	0.730	0.561**	1					
Pride	0.686	0.688	0.514**	0.525**	1				
Connection	0.720	0.716	0.513**	0.458**	0.418**	1			
PE	0.826	0.849	0.600**	0.554**	0.530**	0.522**	1		
PB	0.817	0.816	0.627**	0.603**	0.602**	0.452**	0.602**	1	
EWOM	0.867	0.861	0.602**	0.595**	0.472**	0.545**	0.659**	0.574**	1

CR, construct reliability; EWOM, Employees’ word-of-mouth referrals.

**p < 0.01.

## Study 3

### Sample and procedure

This study uses Credamo and social networks to collect data. This online survey method overcomes the distance barrier and makes data collection more convenient and straightforward. In May 2022, a total of 507 questionnaires were obtained, after excluding 83 invalid questionnaires, a total of 424 valid questionnaires were finally collected for further analysis. The sample used in this study was the same as Sample 2.

The questionnaire used in this study included three sections. The first part clarifies the purpose of the research and core concepts, as well as makes a commitment to data confidentiality to the participants. The second part is the measurement items, where participants were asked to answer the questions carefully about elevation, insight, pride, connection, PE, JR, PB, and employees’ WOM referrals. The third part collects the demographic information of the participants. All participants had work experience in the past month, and all data were collected through employee self-assessment. Moreover, the data and the hypothesized relationships in this study were analyzed using SEM and with the statistical software SPSS and AMOS.

### Measures

#### Measurement of trigger events

This study used self-designed scales to measure elevation (α = 0.717), insight (α = 0.730), pride (α = 0.688), and connection (α = 0.716). All of them were measured on a seven-point Likert scale (1 = strongly agree, 7 = strongly disagree). The specific content of the scale can be found in Study 2.

#### Peak experience

Our research defines PE as a high-activated positive mood, which is a highly positive feeling. Therefore, according to the research of Warr (1990), we selected four items to measure PE: “enthusiastic”, “excited”, “inspired” and “joyful.” We asked respondents to indicate the extent to which they experienced the above emotions in their work experience over the last month (1 = strongly agree, 7 = strongly disagree) (Ouyang et al., 2019). Reliability was acceptable (α = 0.849).

#### Employees’ word-of-mouth referrals

Employees’ WOM referrals were measured using an adapted three-item scale developed by Price and Arnould (1999), and we changed “this hairstylist” to “this organization.” The three items were “I would recommend this organization to someone who seeks my advice”, “I say positive things about this organization to other people”, and “I would recommend this organization to others” (1 = strongly agree, 7 = strongly disagree). Reliability was acceptable (α = 0.816).

#### Proactive behavior

Proactive behavior (PB) was measured using the scale developed by Frese et al. (1997). The seven items were “I actively attack problems”, “Whenever something goes wrong, I search for a solution immediately”, “Whenever there is a chance to get actively involved, I take it”, “I take initiative immediately even when others don’t”, “I use opportunities quickly in order to attain my goals”, “Usually I do more than I am asked to do”, and “I am particularly good at realizing ideas” (1 = strongly agree, 7 = strongly disagree). Reliability was acceptable (α = 0.861).

#### Job relevance

In this study, we want to understand the impact of JR on the relationship between EPE and employee behavior. More specifically, we want to explore what will happen to employees’ PB and employees’ WOM referrals when their triggers of PE are more relevant to the job or not. An item for measuring JR was developed according to the definition and the classification standards of motivational factors and hygiene factors. This item is “the triggers that lead me to have a strong “enthusiastic”/“excited”/“inspired”/“joyful” emotion are mostly related to the work.”

#### Control variable

In order to accurately measure the influence of independent variables on dependent variables, this study takes the respondents’ gender, age, education, tenure, job, and organization to which they belong as control variables.

### Statistical analyses

In this study, data were analyzed using SPSS 26.0 and AMOS 26.0 to test the hypotheses presented in Study 1. First, we describe the data and calculate the mean, standard deviation, and correlation of each variable. Secondly, we test the reliability and validity of the data, analyze the reliability of the measurement tools, and calculate the α, CR, and convergent validity of each variable. Thirdly, maximum likelihood estimation was used to conduct CFA on the observed model, indices such as χ^2^/df, CFI, SRMR, and RMSEA were calculated to test the fit of the model to the data. Finally, path analysis was used to calculate standardized parameter estimates, standard errors, and *p*-values to verify the influence of elevation, insight, pride, and connection on PE, as well as the influence of PE on PB and employees’ WOM referrals. To examine the moderating influence of JR and analyze the impact mechanism of PE on employee behavior, the PROCESS of SPSS was adopted.

## Results

### Descriptive statistical analysis

The indicators of descriptive statistics include minimum, maximum, mean, standard deviation, kurtosis, and skewness, where the mean represents the central tendency of the data, the standard deviation shows the degree of dispersion of the data, and kurtosis and skewness are used for testing whether the data conforms to a normal distribution. The descriptive statistical analysis of each variable is shown in Table 8.

**TABLE 8:** Descriptive statistics.

Statistic	Minimum	Maximum	Mean	SD	Skewness	Kurtosis
Elevation	1.600	7.000	5.379	0.855	−1.118	1.836
Insight	1.250	7.000	5.847	0.702	−1.520	5.260
Pride	3.000	7.000	5.938	0.629	−1.261	2.562
Connection	2.330	7.000	5.769	0.814	−1.133	1.782
PE	1.500	7.000	5.547	0.941	−1.314	2.133
PB	2.710	7.000	5.685	0.686	−1.029	1.199
EWOM	1.000	7.000	5.726	1.018	−1.921	5.038

The correlations between the variables were summarized as follows. Elevation was significantly positively correlated with PE (*r* = 0.600, *p* < 0.01), PB (*r* = 0.627, *p* < 0.01), and employees’ WOM referrals (*r* = 0.602, *p* < 0.01). Insight was significantly positive correlated with PE (*r* = 0.554, *p* < 0.01), PB (*r* = 0.603, *p* < 0.01), and employees’ WOM referrals (*r* = 0.595, *p* < 0.01). Pride was significantly positively correlated with PE (*r* = 0.530, *p* < 0.01), PB (*r* = 602, *p* < 0.01), and employees’ WOM referrals (*r* = 0.472, *p* < 0.01). Connection was significantly positively correlated with PE (*r* = 0.522, *p* < 0.01), PB (*r* = 0.452, *p* < 0.01), and employees’ WOM referrals (*r* = 0.545, *p* < 0.01). PE was significantly positively correlated with PB (*r* = 0.602, *p* < 0.01) and employees’ WOM referrals (*r* = 0.659, *p* < 0.01).

### Reliability and validity analysis

Since we collected data from the participants at one time, there may be a common method variance (CMV) problem in this study. Therefore, we used Harman’s single factor test in SPSS and found that the maximum factor variance explained rate was 36.47%, which was less than 40%, indicating that there was no serious common method bias.

This study also tested the reliability and validity of the items. First, the internal consistency of the items was tested by calculating Cronbach’s alpha. This index in this study varies from 0.688 to 0.861, showing that there is a strong consistency among the items (Nunnally, 1978). Second, the items used in this study were partially derived from widely recognized research and the remaining items were developed following standard procedures, which supported the face validity. Finally, the convergent validity is evaluated by calculating the composite reliability. This index in this study is between 0.686 and 0.729, indicating that the research model has good convergent validity.

In addition, we also tested the variable inflation factor (VIF) of the triggers. We found that the VIF of elevation, insight, pride, and connection were 1.772, 1.699, 1.562, and 1.469, respectively, which were all less than 4.0, indicating that there was no multicollinearity problem (Hair et al., 2018).

### Structural equation modeling

In this study, the method of structural equation modeling (SEM) was used to test the relationship between constructs. Before testing hypotheses, this study conducted a CFA of the model using the maximum likelihood estimation method. The fitting indicators of the model are as follows: χ^2^ = 884.735, df = 393, χ^2^/df = 2.251, RMSEA = 0.054, CFI = 0.904, IFI = 0.905, TLI = 0.894, which satisfy the model fitting conditions (Bollen, 1989; Hu and Bentler, 1999; Kline, 2005; Hair et al., 2017).

**FIGURE 2:**
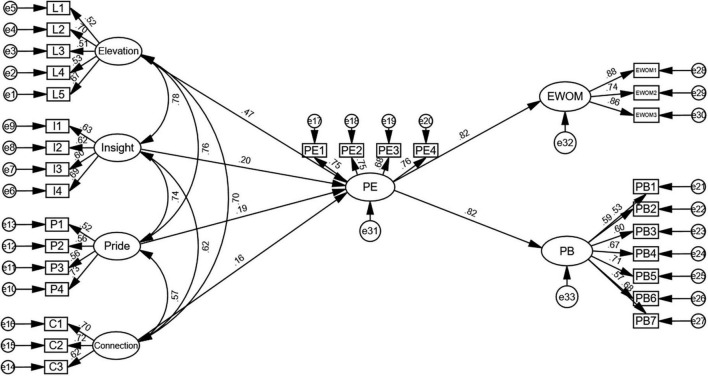
Structural model.

From Table 9, it can be seen that all direct effects are statistically significant. Elevation (β = 0.473, *p* < 0.001), insight (β = 0.202, *p* < 0.05), pride (β = 0.185, *p* < 0.05), and connection (β = 0.155, *p* < 0.05) all have significant positive impact on PE, so H1a–d are supported. PE also has a significant positive effect on PB (β = 0.817, *p* < 0.001) and employees’ WOM referrals (β = 0.823, *p* < 0.001), so H2a,b are supported. Figure 2 shows the relationship between all observable variables and their corresponding latent variables, as well as the relationship between all latent variables.

**TABLE 9:** Standardized parameter estimates, standard errors, and *p*-values for the structural model.

			Estimate	SE	CR	*P*
PE	< –	Elevation	0.473	0.138	4.188	***
PE	< –	Insight	0.202	0.131	2.291	0.022
PE	< –	Pride	0.185	0.136	2.231	0.026
PE	< –	Connection	0.155	0.068	2.424	0.015
PB	< –	PE	0.817	0.070	14.863	***
EWOM	< –	PE	0.823	0.050	9.651	***

***p < 0.001.

### Moderation analysis

In this study, we also used the PROCESS of SPSS macro program model 14 to explore the moderating effect of JR on the relationship between PE and PB, as well as the relationship between PE and employees’ WOM referrals. It can be seen from Table 10 that the interaction between PE and JR has a significant impact on PB (β = 0.062, *p* < 0.001). This indicates that JR can moderate the relationship between PE and PB. Therefore, H3 is supported.

**TABLE 10:** Results of the moderating effect.

	Coeff	SE	*T*	*P*
Constant	5.6518	0.0277	203.9477	0.0000
PE– > PB	0.4044	0.0341	11.8604	0.0000
JR– > PB	0.1363	0.0311	4.3862	0.0000
PE × JR– > PB	0.0620	0.0180	3.4506	0.0006

**FIGURE 3:**
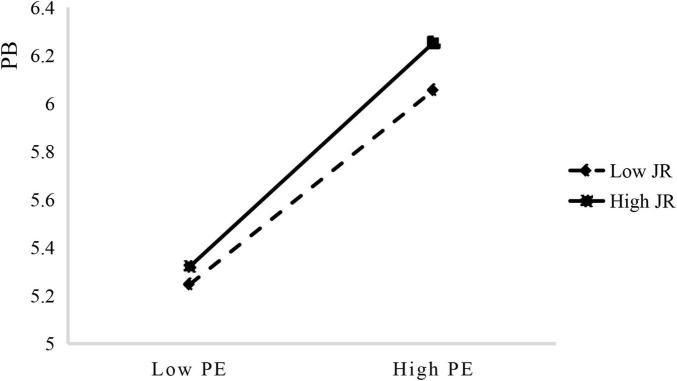
The moderating effect of job relevance (JR) on the relationship between peak experience (PE) and proactive behavior (PB).

To further analyze the effect of JR on the relationship between PE and PB, a graph (Figure 3) was created to show the moderating effect of JR at different levels. When JR is at a low level (M–1SD), PE and PB have a significant correlation (β = 0.339, *p* < 0.001). When JR is at a high level (M + 1SD), PE and PB also have a significant correlation (β = 0.470, *p* < 0.001). It indicates that the more job-relevant the triggers are, the stronger the association between PE and PB is. In other words, JR positively moderates the relationship between PE and PB. Thus, H3 was further supported.

### Mediation analysis

In order to verify the mediating effect of PE, PROCESS of SPSS macro program model 4 was applied. The mediating effect of PE was tested using the bootstrapping method, with a sample size of 5,000 and a bias correction confidence interval of 95%. The test results are shown in Table 11, and all of the confidence intervals did not include 0. Thus, it was verified that PE had mediating effects on four triggers (elevation, insight, pride, and connection) and two behaviors (PB and EWOM). Hypotheses H4a–h were supported.

**TABLE 11:** Results of the mediating effect.

	Effect	Coeff.	SE	Bootstrap 95%	
				LLCI	ULCI
Elevation– > PE– > PB	Direct effect	0.334	0.058	0.218	0.446
	Indirect effect	0.170	0.035	0.105	0.241
	Total effect	0.503	0.048	0.412	0.599
Elevation– > PE– > EWOM	Direct effect	0.383	0.082	0.225	0.551
	Indirect effect	0.333	0.056	0.229	0.445
	Total effect	0.716	0.068	0.579	0.849
Insight– > PE– > PB	Direct effect	0.380	0.070	0.247	0.517
	Indirect effect	0.209	0.042	0.131	0.295
	Total effect	0.590	0.051	0.492	0.691
Insight– > PE– > EWOM	Direct effect	0.480	0.090	0.298	0.653
	Indirect effect	0.383	0.066	0.261	0.525
	Total effect	0.863	0.075	0.716	1.010
Pride– > PE– > PB	Direct effect	0.430	0.067	0.299	0.558
	Indirect effect	0.227	0.041	0.151	0.314
	Total effect	0.657	0.057	0.545	0.766
Pride– > PE– > EWOM	Direct effect	0.277	0.104	0.075	0.479
	Indirect effect	0.488	0.082	0.343	0.661
	Total effect	0.765	0.080	0.614	0.925
Connection– > PE– > PB	Direct effect	0.159	0.062	0.038	0.281
	Indirect effect	0.221	0.041	0.145	0.310
	Total effect	0.380	0.045	0.293	0.471
Connection– > PE– > EWOM	Direct effect	0.346	0.090	0.166	0.521
	Indirect effect	0.336	0.063	0.221	0.470
	Total effect	0.682	0.073	0.538	0.824

## Discussion

Our research responds to the scholarly call to explore how to improve EX, which is under-investigated. Drawing on the increasing attention of high-activated positive mood, this research pays special attention to EPE. In our research, we sought the issue of how to create PE for employees and how these experiences influence their behavior through three studies.

In Study 1, we constructed a theoretical model centered on EPE using a grounded theory approach based on an analysis of semi-structured interview data. This model shows the triggers and impacts of EPE in real work situations, and the connotations and dimensions of these main categories were also explained. This model was constructed on the real work experience obtained through interviews, and it extends the findings of Heath and Heath. Specifically, triggers in this model are consistent with the four-element conceptual model proposed by Heath and Heath (2017), but impacts in this model are related to the behavior of employees, which extends the scene and connotation of the findings of Heath and Heath in the field of HRM.

In Study 2, we developed and validated an instrument to quantify the triggers of EPE using standardized procedures and principles. The scale consists of four subscales that measure four factors: elevation, insight, pride, and connection. It has been proven that the scale is a reliable and valid instrument by testing its reliability and validity in our research. Previous research mostly focused on how to classify the triggers of PE (Kim and Wang, 2020). Our research not only indicated the types of triggers but also developed an effective scale to measure these triggers. The scale is the first instrument to be used to quantitatively measure the triggers of EPE, and it has also been proven to be effective in the testing of variable associations.

In Study 3, we used the scale developed in Study 2 to test the theoretical model proposed in Study 1. In terms of triggers, it is shown that elevation, insight, pride, and connection all have significant positive impacts on PE. Specifically, elevation has a large effect (β = 0.473, *p* < 0.001) on PE because out-of-the-ordinary events do not frequently occur in daily work experience, and participants can easily recall these kinds of events due to their uniqueness. For example, changing work is rarely experienced in daily work, this experience represents a milestone in the employee’s career and leaves a deep impression on them. The effects of insight (β = 0.202, *p* < 0.05) and pride (β = 0.185, *p* < 0.05) are positive on PE, indicating that helping employees gather important understanding and create opportunities to demonstrate their best can let them experience highly positive emotions. The positive relationship between insight and PE can be supported by the findings of Khoi et al. (2021), which prove that suddenly discovering new realizations has an influence on emotional responses of delight. The same conclusion can be summarized from the interview data, which demonstrate that employees can naturally experience positive emotions when they recognize the significant impact of making a breakthrough as well as gaining growth through reflection. In addition, our research expands on the findings of Kim and Wang (2020), which discover finding a sense of self-existence at work is one of the antecedents of PE. The connotations of pride in our research not only include this type of event but also contain other positive events that can help employees show their excellent ability, such as getting praise or appreciation from others (Ohly and Schmitt, 2013), as well as being able to handle a challenging situation (Tavel et al., 2022). All of these positive work events can trigger employees’ positive emotions because they are their moments of glory (Yang et al., 2022). Moreover, connection has a positive but small direct effect (β = 0.155, *p* < 0.05) on PE. While companionship plays a key role in shaping memorable experiences (Vada et al., 2022), when employees are in trouble, getting timely and effective help can shed light on their hardship (Paakkanen et al., 2021). These things can make employees realize that they are closely bonded with their co-workers and can make them have memorable experiences. The reason why the effect of connection and PE is smaller than other triggers between PE is probably that the measurement items we used are more focused on the employee’s initial stage, where some help and welcome at that time may be taken for granted by some participants.

We also tested the impacts of EPE in Study 3. According to existing studies, employees’ behaviors are closely related to emotions (He and Wei, 2022). Findings about high-activated positive mood (e.g., enthusiastic, inspired, and excited) have also indicated that positive emotions can lead to positive behavior (Mindeguia et al., 2021). As PE is a kind of high-activated positive mood, we believed that PE can promote employees’ behavior. In line with this proposition, we found that employees who have PE are more likely to make good WOM referrals and adopt PB. Specifically, PE, as hypothesized, has a positive, significant, and very large effect on PB and WOM referrals with coefficients of 0.817 (*p* < 0.001) and 0.823 (*p* < 0.001), respectively. This result reinforces the idea that when employees have highly positive emotions, there is an increase in the probability of their behaving in a way that is beneficial to the organization. In addition, we further examined what kinds of factors can motivate employees to work hard. We found that JR could positively moderate the relationship between PE and employees’ PB (β = 0.062, *p* < 0.001). When JR is at a high level, PE and PB have a stronger significant correlation (β = 0.470, *p* < 0.001) than JR is at a low level (β = 0.339, *p* < 0.001). This phenomenon can be explained based on the two-factor theory, motivational factors are largely related to work, and those factors can stimulate employees’ enthusiasm and make them generate excitement and energy (Liu et al., 2021), thus making employees more proactive at work.

## Theoretical contributions

First, our research constructed a theoretical model centered on EPE. Existing research on PE is mainly focused on CX, and our model is the exploration of EX. This model was constructed using a grounded theory method, which also verified the findings of Heath and Heath in the field of HRM. We found that triggers including elevation, insight, pride, and connection can stimulate the PE of employees, and this highly positive EX can positively affect employees’ behaviors like PB and WOM referrals.

Second, our research solved the problem of how to measure the triggers for EPE. Previous research paid more attention to summarizing the types of triggers for EPE, and we have little knowledge about how to use them in empirical studies. Thus, we developed a scale to measure the triggers, and the initial items came from interview data. This scale contains four subscales to measure elevation, insight, pride, and connection, respectively, and its reliability and validity have also been demonstrated in our research.

Finally, our research verified the theoretical model using questionnaire data, which has not appeared in previous works about EPE. Almost all of the existing research on PE relies merely on qualitative interviews to identify the triggers and impacts. We combined qualitative and quantitative methodologies to further investigate PE. In our research, we used the scale developed by ourselves to test the model that focuses on EPE and verified it using questionnaire data.

## Managerial implications

On the one hand, our research puts forward some implementable measures for organizations to create PE for their employees. Currently, in the field of HRM, an increasing number of managers are devoted to creating positive EX. PE, which is a highly positive EX, will bring more benefits to organizations. The scale developed in our research provides an easy-to-understand and user-friendly tool for organizations to create PE for their employees by focusing on four types of positive work events: elevation, insight, pride, and connection. For example, giving employees a certain degree of decision-making power or providing employees with some high-value benefits are measures to create a PE for employees in the category of elevation. Providing training opportunities that are conducive to employees’ growth and development is a measure from the insight category to create PE for employees. Giving full recognition and affirmation of the employees’ work attitude and performance are measures to create a PE for employees in the category of pride. Creating a mutually helpful, harmonious, and friendly working atmosphere is a measure from the connection category to create PE for employees. Thus, focusing on the triggers of EPE can put forward improvement measures to help organizations and promote them to develop in a better direction.

On the other hand, our research emphasizes the importance of designing EPE for organizations. After organizations create EPE, their employees will not only make more positive WOM referrals but also adopt more PB. Therefore, creating PE for employees can help organizations build a good image as well as motivate employees to contribute and work harder. Moreover, if organizations want to make employees work more actively and efficiently, they should pay more attention to creating EPE whose triggers are relevant to the job, such as praising their employees. If organizations want to increase positive WOM, they should make every effort to create as much EPE for their employees as possible, regardless of whether the triggers are relevant to the job.

## Limitations and future directions

There are several limitations to our research. First, our research uses data that was collected at a certain time and was self-reported by the participants to test the theoretical model. Although we have proven that there is no CMV problem, researchers can increase the accuracy by using multi-source data and longitudinal methodology. Second, other moderating and dependent variables should be investigated. The moderating influence of some individual factors on the relationship between constructs can be explored by researchers. For example, after experiencing the same PE, extroverted employees may be more likely to make positive WOM referrals than introverted employees. Similarly, researchers can also investigate the impact of PE on employee performance and employee engagement. Third, our research did not limit the situations in which PE occurred when interviewing the triggers and impacts of PE. Therefore, the triggers and impacts of EPE can be explored more specifically in the future, such as in interpersonal collaborative work situations or recruitment stages, and the scale developed in our research can be tested in new work situations.

## Conclusion

We find that the triggers that enable employees to generate EPE include elevation (moments of transcending ordinary events), insight (moments of rewiring one’s understanding), pride (moments of capturing one’s best), and connection (moments of being bonded with others). Employees will experience PE if they perceive at least one of the factors above at work.

We also discovered the impacts of EPE. According to our research, employees that have PE are more likely to make good WOM referrals and adopt PB. Four triggers (elevation, insight, pride, and connection) positively predict employees’ WOM referrals and PB through the mediating role of PE. We also found that JR could moderate the relationship between PE and PB. Specifically, the more job-relevant the triggers are, the more PB employees will engage after they have EPE.

Moreover, we developed and validated a scale for measuring the triggers of EPE, which contains four subscales with a total of 16 items. Based on this scale and other tools, the causal relationship of EPE proposed by theoretical model can be proven using data in our research.

## Data availability statement

The raw data supporting the conclusions of this article will be made available by the authors, without undue reservation.

## Ethics statement

The studies involving human participants were reviewed and approved by Ethics Committee of Donghua University. The patients/participants provided their written informed consent to participate in this study.

## Author contributions

XF provided efforts in conceived the idea, data collection, review, and provided quality assurance of the research. JM was responsible for conducting the interviews, performing the data analysis, interpreting the data, and drafting the manuscript. Both authors contributed to the conceptualization, design of the research manuscript, and approved the submitted version.
